# The Beat

**Published:** 2010-01

**Authors:** Erin E. Dooley

## Persistent Contaminants in Newborns

In December 2009 the Environmental Working Group (EWG) released *Pollution in People: Cord Blood Contaminants in Minority Newborns*, which presents data on chemicals found in the cord blood of 10 newborns. The report includes what the authors call the first reported detection of 21 synthetic compounds in cord blood, including bisphenol A in 9 of the babies. The goal of this and similar EWG reports is to quantify the number of pollutants found in people, with 414 chemicals detected to date. The report is available at http://www.ewg.org/.

## Outdoor Smoking: Minor Respite

As more jurisdictions around the world implement bans on smoking in indoor settings, many bars and restaurants are accommodating smoking patrons with outdoor smoking areas. Now a report in the November 2009 issue of the *Journal of Occupational and Environmental Hygiene* says exposure to secondhand smoke (SHS) in outdoor seating areas may affect the health of waitstaff and bouncers who may be exposed for several hours a day. Luke Naeher and colleagues stationed nonsmoking volunteers for 6 hours in outdoor bar or restaurant seating or outside a college library and found their salivary levels of cotinine increased over baseline by 162%, 102%, and 16%, respectively. Although cotinine levels were relatively low compared with levels following indoor exposure to SHS, the U.S. Surgeon General has determined there is no safe level of exposure to cigarette smoke.

**Figure:**
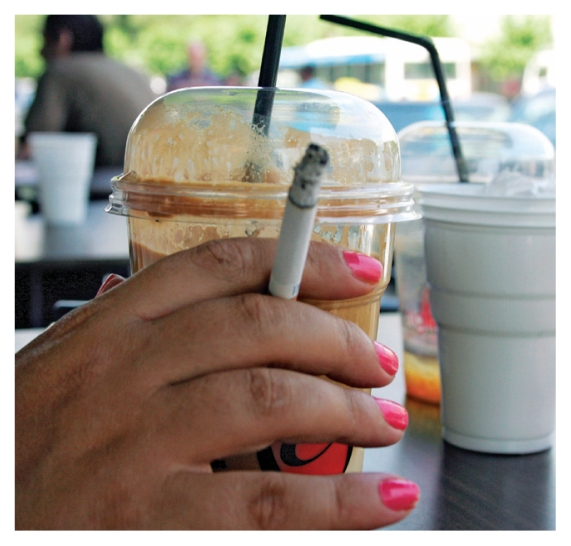
Relegating smoking areas to outdoors may not fully prevent SHS exposure

## On-the-Job Exposure to Endocrine Disruptors

Martijn M. Brouwers and colleagues have revised an existing job exposure matrix to more accurately assess potential occupational exposures to endocrine disruptors. As outlined in the September 2009 issue of *Occupational and Environmental Medicine*, the updated matrix can be adjusted to allow for more widespread use with specific jobs and tasks. Among other changes, the authors incorporated information on recently identified endocrine disruptors and converted dichotomous exposures scores (yes versus no) with exposure probabilities of low, medium, and high.

## Sick Schools Revisited

In December 2009, the National Coalition for Healthier Schools released an updated report on environmental health factors in U.S. schools. *Sick Schools 2009*, available at http://www.healthyschools.org/, gives a state-by-state illustratration of how poor air quality in some schools increases health care costs and absenteeism while negatively impacting test scores. Overall, the report estimates 57% of U.S. public school students attend schools with at least one “unsatisfactory environmental factor.” The authors recommend full staffing and resources for the U.S. EPA and other federal agencies to address healthy school environments.

**Figure:**
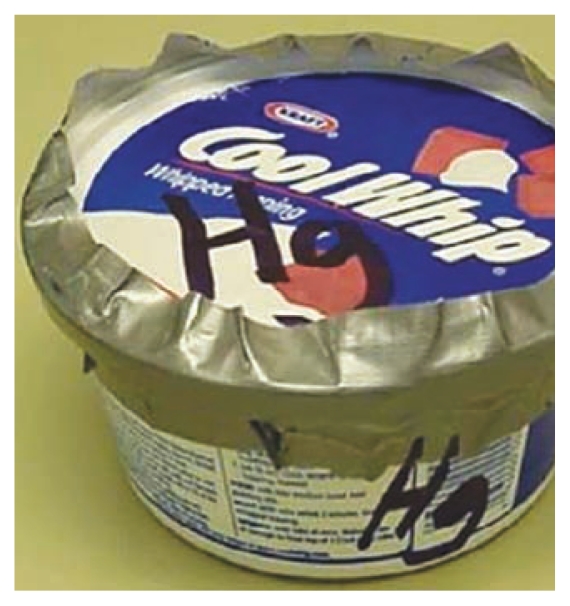
Science class storage: what not to do

## Scandinavian Cell Phone Study: No Brain Tumor Association

A 30-year Scandinavian study by Isabelle Deltour and colleagues published online 3 December 2009 in the *Journal of the National Cancer Institute* found no change in brain tumor incidence trends between 1998 and 2003. Widespread cell phone use began in Scandinavia in the mid 1990s; the authors write that changes in brain tumor rates after 1998 “would be informative about an induction period of 5–10 years.” They add the caveat that it may take longer than that for tumors caused by cell phone use to be detected.

## EPA Proposes 1-Hr SO_2_ Standard

Short-term exposures to sulfur dioxide can cause a variety of respiratory symptoms and increased hospital admissions. The EPA’s current sulfur dioxide 24-hr primary standard of 140 ppb and annual average standard of 30 ppb have been on the books since 1971. The agency is now accepting comments to develop a more protective 1-hr primary standard of between 50 and 100 ppb, which would replace the existing standards. The EPA is also discussing changes to monitoring and reporting requirements for the pollutant, two-thirds of which comes from coal-fired power plants. The EPA expects to issue the final standard by June 2010.

**Figure:**
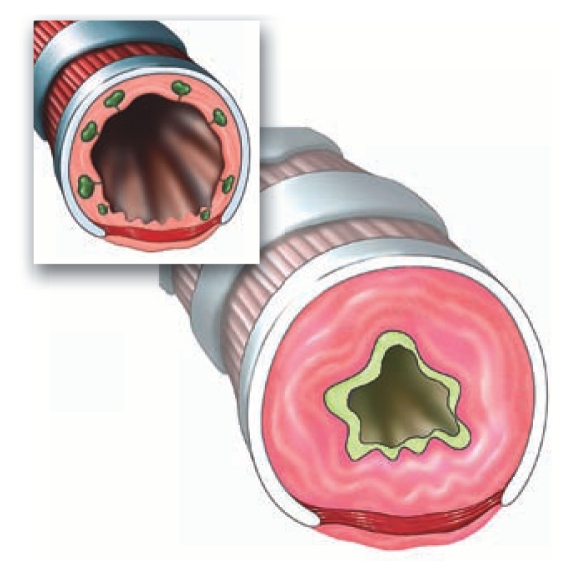
SO_2_ constriction, triggering asthma attacks (inset: normal bronchiole)

